# New Hampshire Veterinary Medical Association

**Published:** 1897-01

**Authors:** L. Pope

**Affiliations:** Secretary


Secretary,

### NEW HAMPSHIRE VETERINARY MEDICAL ASSOCIATION.

The thirteenth meeting was held at the Eagle Hotel, Concord, on Tuesday, December 8th, at 11.30 A.M., with Dr. Lilico in the chair. Drs. Lilico, Tuttle, Hart, Abbott, Russell, Macguire, and Pope responded to roll-call.

Dr. Pope read a report on the proceedings at the United States Veterinary Medical Association Convention at Buffalo in September.

Dr. Lilico, chairman of the Legislative Committee, presented a proposed bill from the committee to go before the coming Legislature. The bill was discussed by sections and a few changes suggested. Dr. Lilico was then authorized to employ counsel and have the bill put into proper shape and the penalties attached. After its completion, the Secretary was instructed to call a meeting to reconsider the changes made. Dr. R. J. Macguire then read a most interesting paper on "Abortion." Meeting adjourned to March.

L. Pope, Jr., M.D.V.,
Secretary.

